# Introduction to the Topic of the Special Issue “Progresses and Challenges of Block Copolymer Membranes” from the Guest Editor

**DOI:** 10.3390/membranes13070687

**Published:** 2023-07-24

**Authors:** Ilsiya M. Davletbaeva, Oleg O. Sazonov

**Affiliations:** Technology of Synthetic Rubber Department, Kazan National Research Technological University, 68 Karl Marx Street, Kazan 420015, Russia; sazonov.oleg2010@gmail.com

It is well-known that various mixtures that require separation have to be dealt with in many branches of industry, especially the chemical and petrochemical industries. Mixture separation technologies are in great demand and cannot be dispensed with. A mixture is a system consisting of several components; for example, seawater, air, and various emulsions are all mixtures. Mixtures are either heterogeneous or homogeneous. The difference between them lies in the fact that heterogeneous mixtures are a combination of several substances that differ in their state of aggregation. Homogeneous mixtures are homogeneous liquid systems and mixtures of gases. Evaporation, distillation, and crystallization are used to separate liquids. For gases, there are methods of condensation and absorption, as well as processes associated with chemical transformations. There is a wide range of disadvantages of traditional separation methods; each method has its own limiting factors, but the most common are metal consumption, high-energy consumption, and the speed and selectivity of the process. Because of these disadvantages, more advanced separation methods are being created; the most advanced of which are membrane technologies.

A membrane is a semi-permeable barrier capable of selectively allowing molecules of one substance to pass through and blocking or retaining molecules of another type. In membrane technologies, it is necessary to create the driving force of the process, because this ensures mass transfer. One of the most important characteristics of an isolating diaphragm is its selectivity. It is a dimensionless unit indicating the efficiency of membrane separation under the action of a driving force in the system to be separated. This value can be infinitely large, and the larger it is, the better, due to the fact that the membrane separation process is not ideal, and some by-products can pass through our partition along with the target product; the lower this value is, the higher the selectivity. The performance of the membrane, or its throughput, shows how much of the substance the membrane can allow to pass through per unit of time. At the same time, it is worth mentioning membranes that work according to the diffusion mechanism, i.e., non-porous and, according to the Knudsen mechanism, porous. In total, at the moment there are several main types of membranes that are widely used in industry.

Porous membranes include microfiltration, ultrafiltration, and reverse osmosis membranes. They are used to separate substances for which it is sufficient to achieve a pore size in the range of 100 nm to 2 nm. The microfiltration membrane is a filter that retains particles from 0.1 to 10 microns. A porous structure can be achieved via sintering polymer granules such as ultra-high molecular weight polyethylene by stretching a polymer web to form holes of a given size, which is achieved by penetrating the material with heavy particles. Ultrafiltration membranes are another type of porous membrane that occupy an intermediate position between microfiltration and reverse osmosis membranes. The size of the particles retained by this type of membrane ranges from 1 to 100 nm, and such a membrane is already capable of separating various bacteria, fats, proteins, and other organic substances. In general, such polymers are most often obtained using the phase inversion method. Reverse osmosis membranes are used in the separation of solutes in a solvent, and the separation of organic salts or glucose from water. The nanopore size of such coatings is less than two nanometers.

Gas separation membranes already work at the molecular level. In nature, the main mechanism of mass transfer is diffusion, in which the gas is dissolved in the volume of the polymer, followed by its “pulling” from the polymer matrix and desorption. Gas separation can also be carried out using the Knudsen diffusion mechanism, which is when mass transfer occurs through pore channels. An increase in the number of gas collisions with neighboring molecules and with the channel walls leads to the termination of the gas transport process. Gas separation membranes are used in the separation of a mixture of gases in the synthesis of ammonia, for concentrating gases, purifying methane, extracting hydrogen, and so on. To evaluate the effectiveness of the membrane, the Robson curve is used. The closer the point is to the Robson curve, the more effective the membrane. Thus, according to the Robson curve, high selectivity values are accompanied by low membrane productivity, calculated in this case in Barrers.

Pervaporation membranes are based on the diffusion mechanism of mass transfer, which includes selective sorption, subsequent diffusion and desorption of the separated component through a non-porous membrane. Pervaporation is used primarily to separate liquid substances capable of forming azeotropes. This technology allows us to separate mixtures of water and alcohol, solvents, and even aromatic and aliphatic compounds. To intensify diffusion processes, pervaporative membranes must be amorphous. The limiting swelling of the membrane in the medium, the thickness of the membrane, its thermal stability, and its resistance to the medium being separated are important. Polyurethanes, polydimethylsiloxanes, polytetrafluoroethylenes, polyphenylsulfones, polyvinyl alcohol, which initiated this type of membrane, and many others are used as pervaporation membranes.

To create an effective membrane, many factors must be considered. In addition to the composition of the polymer itself, the order, combination, and sometimes the location of the same link within the chain matters. The cross-linking of the polymer has a significant effect on mass transfer, which hinders the diffusion of the separated substances, but at the same time ensures the mechanical properties of the membrane. It is known that linear polymers are prone to coiling, through which it is almost impossible for an individual molecule of the substance to be separated to push through. Therefore, partially cross-linked polymer structures are quite common. The mass transfer of gases and vapors through membranes also depends on the state of the polymer. In general, a polymer in a glassy state has a low permeability compared to a highly elastic polymer. The molecular weight of the polymer is just as important; for example, high-molecular-weight polymers are more likely to be entangled than low-molecular-weight polymers, which has a direct impact on the amount and quality of material that can be forced through the membrane.

Along with synthetic approaches, the mixing of two different homopolymers is used to obtain new polymeric membrane materials. One of the most serious problems here is associated with phase separation due to the low entropy of mixing polymers with different chemical structures. The main way to increase the thermodynamic compatibility of polymers is the synthesis of block copolymers. This strategy has helped create materials with improved mechanical, thermal, and other properties.

Block copolymers, which are the objects of study in the articles related to this Special Issue “Progresses and Challenges of Block Copolymer Membranes”, stand out in a specific category among polymeric materials used in membrane technologies and represent a class of polymers formed by two or more homopolymer fragments connected with covalent bonds. Due to their common mutual insolubility, chemically different blocks readily form phase-separated intermolecular structures, either in a solid state or in a solution. One of the features of block copolymers is their ability to form many ordered nanosized structures. The size, nanoscale periodicity, order, and orientation of such microphases in the solid state can be controlled by precisely varying the molecular weights of blocks, molecular weight distribution, composition, and interaction parameters between block components, and reaction conditions. The macromolecular architecture is a key factor controlling both the resulting morphologies, including spherical, cylindrical, lamellar, and others, and their degree of long-range order. Thanks to these interesting features, research on block copolymers has become popular all over the world.

The synthesis of block copolymers is an established field with many major advances. However, efforts in the field of controlled polymer synthesis allow for a wide range of new block copolymer architectures, including linear, graphic, dendritic, star-shaped, brush, hyperbranched, and cyclic block copolymers, with unique and interesting self-assembly behavior.

There are numerous routes for the synthesis of block copolymers of various structures and compositions, which include the sequential addition of monomers via living anionic polymerization techniques, controlled radical polymerization, macroinitiation, and coupling reactions exploiting the active chain ends of different chain segments. These methods are based on the possibility of obtaining polymers of a certain chemical structure with controlled dispersity, high purity, and the accuracy of inclusion of special functional groups. Each of these factors is of key importance for the perfection of the processes of the self-assembly of macromolecules. Due to macromolecular equivalence and end group accuracy and the problems of premature completion of the synthesis or inefficient binding, the mixture may contain homopolymer or other “incomplete” contaminants. Overcoming these limitations will make it easier to obtain sustainable materials, as well as provide opportunities for the high-performance synthesis of complex architectures.

Advances in the synthesis, functionalization, processing, and characterization of polymers have created a salutary opportunity to design, manufacture, and study a wide range of block copolymers with diverse and complex self-assembly potential in bulk, thin film, and dilute solutions. Thus, self-assembly in a block and the phase behavior of traditional diblock copolymers are the most studied, both theoretically and experimentally.

It seems to us that, at this point in time, the following problems can be singled out, as they are in some way related to the use of block copolymers in membrane technologies.

*The processes of the self-assembly of block copolymers in thin films*, *the thickness of which is several hundred nanometers.* In this case, in addition to the factors affecting the assembly in volume, the self-assembly processes are strongly influenced by the surface energy, the film thickness, and the history of their heat treatment. The listed effects and kinetic limitations associated with the assembly of thin films have a significant effect on the formation of the nanostructure and surface topology in thin films. Controlling the morphology of thin films of block copolymers turned out to be the most relevant in the development of new ultrafiltration membranes.

*The formation of block copolymers from dilute solutions, including the use of several solvents, as a way to control the processes of the self-assembly of block copolymers.* In this case, interaction parameters and entropy effects are additionally included, which can significantly affect the formation and stability of macromolecular ensembles in solution and, as a consequence, sharply affect the assembly process and the resulting nanostructures.

*Biohybrid block copolymers.* Biohybrids contain at least one biomolecular block structure, which can be used as sugars or peptides. This type of self-assembling material creates the possibility of building hierarchical supramolecular structures based on specific interactions. The combination of synthetic and bioblocks is capable of creating unusual nanostructures that cannot be formed in organo-organic block copolymers.

*The use of compatible solvents for the constituent elements of block copolymers*. Solvent compatibility is of particular importance in the synthesis of block copolymers using macroinitiation and post-polymerization coupling. If this problem is not properly considered, it can be difficult to achieve high yields and the complete formation of block copolymers. Solvent compatibility also becomes important in the synthesis of organic biopolymers, the building blocks of which often have limited solubility in a wide range of solvents.

*Theoretical and modeling methods for significant insights into the assembly of block copolymers.* Theoretical efforts provide information useful in the fabrication of new nanoscale materials based on block copolymers and complement experimental works. Theoretical advances make it possible to predict and establish the components for the synthesis of block polymers, their molecular weights, architecture, dispersion, and density. These possibilities will greatly simplify the synthesis and will provide the desired macromolecular data and the design of macromolecules.

Block copolymer self-assembly processes can be used as a powerful tool for the development of advanced membrane materials. It is known that, when obtaining nanoporous membranes, purposeful control of the self-assembly process is the main problem of creating cylindrical porous structures located perpendicular to the surface. Some studies have shown that di- and tri-block copolymers form different nanopores, and since the pore size is uniform throughout the membrane, these block copolymers are most likely to be the membranes of the future.

The multitude of possible building blocks for the next generation of block copolymers is pushing the limits of nanomaterial performance, reinforcing the urgent need to improve the theoretical and experimental methods used in the development of new materials. The emergence of polymers with complex self-organization enhances the role of thermal prehistory and the influence of external conditions on the assembly of macromolecules. An important condition for successful experimental studies is the use of a full set of tools of synthetic, molecular, and nanostructural methodologies for the complete and accurate characterization of macromolecular ensembles. Thus, using the strengths of chemistry, physics, processing, and the theory of creating complex nanomaterials based on block copolymers, new membrane materials will be developed and the gap between theoretical promises and their practical applications will continue to close.

Given number of works devoted to these interesting and important tasks, the modern approach to the creation of membrane materials based on the synthesis of block copolymers should be much larger compared to its current size. This Special Issue of *Membranes* intends to contribute to the development of the scientific direction associated with these objectives.

